# Highlight—“Junk DNA” No More: Repetitive Elements as Vital Sources of Flatworm Variation

**DOI:** 10.1093/gbe/evab217

**Published:** 2021-10-05

**Authors:** Casey McGrath

“The days of ‘junk DNA’ are over,” according to Christoph Grunau and Christoph Grevelding, the senior authors of a new research article in *Genome Biology and Evolution.* Their study provides an in-depth look at an enigmatic superfamily of repetitive DNA sequences known as W elements in the genome of the human parasite *Schistosoma mansoni* (Stitz et al. 2021). Titled “Satellite-like W elements: repetitive, transcribed, and putative mobile genetic factors with potential roles for biology and evolution of *Schistosoma mansoni*,” the analysis reveals structural, functional, and evolutionary aspects of these elements and shows that, far from being “junk,” they may exert an enduring influence on the biology of *S. mansoni*.

“When we studied genetics at university in the 1980s, the common doctrine was that the non-protein coding parts of eukaryotic genomes consisted of interspersed, ‘useless’ sequences, often organized in repetitive elements like satellite DNA,” note Grunau and Grevelding. Since then, however, the common understanding of such sequences has fundamentally changed, revealing a plethora of regulatory sequences, noncoding RNAs, and sequences that play a role in chromosomal and nuclear structure. With their article, Grunau and Grevelding, along with their coauthors from Justus Liebig University Giessen, University of Montpellier, and Leipzig University, contribute further evidence to a growing consensus that such sequences play critical roles in evolution.

**Fig. 1.**
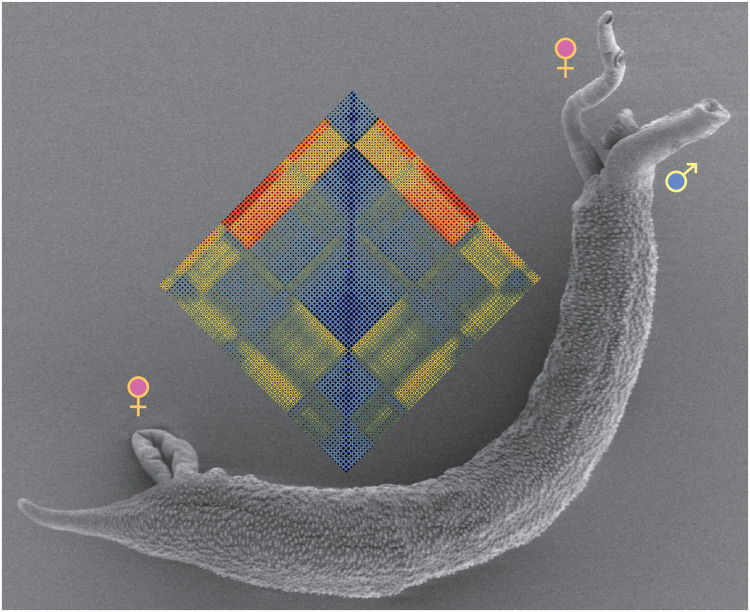
Electron microscopy image of a *Schistosoma mansoni* couple (courtesy of Mandy Beutler). Upon pairing, the larger male clasps the smaller female by its body edges, thus forming a ventral groove in which the female lodges for the continuous pairing contact necessary for female sexual maturation. Sometimes, the anterior and/or posterior ends of the female loop out of the ventral groove, as seen here. The image is overlaid by a sample-distance matrix analysis showing the transcript occurrence of satellite DNA-like W element families transcribed at different levels across larval and adult stages. For more information, see Stitz et al. (2021, Supplementary Fig. 6).

Flatworms such as *S. mansoni* provide a fascinating subject for such a study. They have a complex life cycle, including an asexual reproductive phase that takes place inside an intermediate host—a freshwater snail—and a sexual reproductive phase that takes place inside the human host, where it causes schistosomiasis, a neglected tropical disease that nearly rivals malaria in terms of human morbidity and mortality. Unlike other trematodes that are exclusively hermaphroditic, schistosomes have two separate sexes. Even more unusual is that constant pairing between schistosome partners is required for the sexual maturation of the female (see fig. 1).

Despite this irregular life history, sex in schistosomes is determined chromosomally, with males having two Z chromosomes and females having one Z and one W chromosome. The nonrecombining portion of the W chromosome is composed mostly of repetitive DNA sequences known as W elements. In the past, these sequences were assumed to be nonfunctional and female-specific, such that they were even used as a marker to identify the sex of schistosome larvae.

Grevelding says that it was an accidental observation that first drew his attention to these W elements. “The first W elements were originally found only in the females of Puerto Rican *S. mansoni* isolates. When I started to investigate these W elements in a Liberian strain, it turned out that they also occurred in males. Further studies showed unexpected variation in W elements originating from mitotic recombination during the asexual phase in the snail intermediate host, indicating the presence of W elements on autosomes.” Unfortunately, at the time of this discovery, further investigations were made difficult due to the lack of genomic resources and appropriate techniques in *S. mansoni*.

Recently, however, new genomic and transcriptomic data have allowed Grunau and Grevelding to take a deeper look at this mystery. In their comprehensive analysis of W elements across the genome, the authors identified 19 W element families, varying in copy number from 3 to 450 at one or several locations on the W chromosome. Notably, 15 of these families had related sequences—representing either full-length or partial W elements—on one or more autosomes, with 13 having representatives on all 7 autosomes. This corroborates Grevelding’s earlier studies and suggests that W elements have a mobile nature. Indeed, some of them exhibited similarities to known mobile genetic elements. A comparative analysis across three closely related schistosome species showed several differences in W element occurrence and structure, with most W elements being much shorter in the other species than in *S. mansoni*.

In analyzing transcriptomic data across *S. mansoni* strains, life stages, sexes, and gonad tissues, the authors found a high degree of variability in the expression of W element transcripts. W element expression was highly complex and occurred throughout schistosome development, exhibiting stage-, sex-, pairing-, gonad-, and strain-specific expression patterns. While no protein-coding open reading frames were identified in the W elements, the authors did identify putative functional RNAs, including microRNAs, small nucleolar RNAs, and self-cleaving ribozymes known as hammerhead ribozymes, indicating that these elements can carry genetic information.

Based on their findings, the authors hypothesize that W element presence, location, and copy number may change rapidly over time and across generations. Indeed, they note that variability and genome plasticity are hallmarks of parasite genomes, with different mechanisms giving rise to such variation in different lineages. This variability/plasticity may help parasites escape host surveillance and colonize new host environments. The authors hypothesize that W elements “not only influence the biology of *S. mansoni*, but they might represent one of the sources of heritable variability, thus shaping the evolution of the family Schistosomatidae.”

As Grevelding points out, one of the caveats of this study is that the functional predictions of W elements “are mainly based on bioinformatics analyses and have to be substantiated by functional analyses. This is also true for our hypothesis about the mobile character of W elements, which we concluded from genome and structural analysis, but for which we have no direct functional evidence yet.” He continues, “Studying the functional roles of W elements, which occur at high copy numbers throughout the genome, will require sophisticated transformation techniques targeting several, large chromosomal loci in parallel.” Unfortunately, such studies are currently limited by the molecular and genetic resources available in this species. Grevelding notes that “one of the obstacles of future research is the lack of protocols to genetically manipulate schistosomes and other multicellular parasites by stable transformation.” While CRISPR/Cas9-mediated genome editing is possible in a few parasites, including schistosomes, the technique is still in its infancy in these organisms. Therefore, just as Grevelding’s earlier work awaited new genomic data for corroboration, additional progress in *S. mansoni* manipulation may be needed before the authors’ current hypotheses can be definitively confirmed.
